# Mycophenolate mofetil in giant cell arteritis

**DOI:** 10.3389/fmed.2023.1254747

**Published:** 2023-11-09

**Authors:** Anne Pankow, Sena Sinno, Thorsten Derlin, Marcus Hiss, Annette D. Wagner

**Affiliations:** ^1^Department of Rheumatology and Clinical Immunology, Charité – Universitätsmedizin Berlin, Berlin, Germany; ^2^Department of Nephrology, Hannover Medical School, Hanover, Germany; ^3^Department of Nuclear Medicine, Hannover Medical School, Hanover, Germany

**Keywords:** Mycophenolate mofetil, giant cell arteritis, vasculitis, case series, imaging

## Abstract

**Introduction:**

Giant cell arteritis (GCA) is a systemic granulomatous vasculitis affecting the large arteries. Abnormal lymphocyte function has been noted as a pathogenic factor in GCA. Mycophenolate mofetil (MMF) inhibits inosine monophosphate dehydrogenase and is therefore a highly lymphocyte-specific immunosuppressive therapy. We aimed to assess the efficacy of MMF for inducing remission in GCA.

**Methods:**

Seven patients (5 female, 2 male) with GCA under therapy with MMF and who were treated at the outpatient clinic for rare inflammatory systemic diseases at Hannover Medical School between 2010 and 2023 were retrospectively included in the study. All patients underwent duplex sonography, ^18^F-fluorodeoxyglucose positron emission tomography (^18^F-FDG PET), magnetic resonance imaging (MRI), and/or biopsy to confirm the diagnosis. The primary endpoints were the number of recurrences, CRP levels at 3–6 and 6–12 months, and the period of remission.

**Results:**

All patients in this case series showed inflammatory activity of the arterial vessels in at least one of the imaging modalities: duplex sonography (*n* = 5), ^18^F-FDG PET (*n* = 5), MRI (*n* = 6), and/or biopsy (*n* = 5). CRP levels of all patients decreased at the measurement time points 3–6 months, and 6–9 months after initiation of therapy with MMF compared with CRP levels before MMF therapy. All patients with GCA in this case series achieved disease remission.

**Discussion:**

The results of the present case series indicate that MMF is an effective therapy in controlling disease activity in GCA, which should be investigated in future randomized controlled trials.

## Introduction

Giant cell arteritis (GCA) is a systemic granulomatous vasculitis that predominantly affects large-sized arteries. GCA is one of the most common primary form of vasculitis and has a prevalence of 24–280 cases per 100.000 persons older than 50 years (1). GCAs are associated with significant morbidity. The arterial inflammatory process may involve the aorta, coronary arteries, or vessels of the extremities in addition to the extracranial arteries. The most serious consequences are blindness, aortic aneurysm, myocardial infarction and stroke. Clinically, GCA is characterized by cranial symptoms such as headache and scalp pressure tenderness. In most patients, a systemic inflammatory response is seen with fever, weight loss, night sweats and a rapid response to glucocorticoid therapy (2).

Abnormal lymphocyte function was found as one pathogenic factor in large vessel arteritis (3). Mycophenolate mofetil inhibits the inosinemonophosphate dehydrogenase DNA synthesis pathway, and is therefore a highly lymphocyte-specific immunosuppressive therapy (4). Considering the pathogenesis of GCA, it is apparent that MMF may be a suitable therapeutic approach for the treatment of GCA, nevertheless there is little data on the success of the therapy so as to date.

Despite the lack of randomized clinical trials, the putative crucial role of lymphocytes in the pathogenesis of GCA and the safe, well-tolerated treatment and proven efficacy of MMF in other vasculitis, such as ANCA vasculitis (5), have led to the use of MMF in clinical practice.

Although there are no defined biological markers to assess disease activity, patients with GCA usually have elevated C-reactive protein (CRP) levels. Therefore, normal CRP levels are good indicators of disease remission (6).

The guideline on therapeutic procedures in diagnosed GCA recommends glucocorticoid-sparing therapy with tocilizumab and, if necessary, methotrexate after individual consideration. MMF is not recommended in national guidelines due to lack of randomized trials. To our knowledge, there are currently two published studies on the usefulness of MMF in GCA, however, they were single case studies (7, 8).

## Patients and materials

### Inclusion of patients

The included patients were treated at the outpatient clinic for rare inflammatory systemic diseases at Hannover Medical School between 2010 and 2023 and retrospectively included in this study. Diagnosis was confirmed by typical clinical symptoms, laboratory, duplex sonography, ^18^F-fluorodeoxyglucose positron emission tomography (^18^F-FDG PET), magnetic resonance imaging (MRI), and/or biopsy. Table 1 summarizes the detailed patients characteristics.

**Table 1:** Demographic data, symptoms, diagnostics and therapy for all patients.

Patient, age (years), sex (♀, ♂)	Clinical presentation	Time of diagnosis of GCA	Ultrasound	Biopsy of arteria temporalis
Patient 1, 82/♀	Shortened walking distance, worsening of general condition, swelling of the right thigh with prolonged walking distance, bilateral pain in the calves due to exertion, double vision when looking to the left, temporary hypesthesia of the legs, claudication in the calf area	11/12	11/12 Arterial occlusion of the femoral type with moderate stenosis of the distal superficial femoral artery on the right and higher-grade stenosis at the same site on the left 07/13 Exclusion of carotid stenosis 10/13 Circulation of legs stable, sonographic changes indicating vasculitis appear regressive	n/a
Patient 2, 81/♀	Unilateral headache on the left, intermittent visual disturbances, reduction in general condition, claudication in the upper extremities, severe feeling of illness, blood pressure measurement in the upper extremities not possible	06/17	06/17 ‘Halo sign’ at the exit of the left temporal artery, preauricularly and in the posterior region of the branch of the temporal artery; occlusions of the axillary artery on both sides. 12/17 Longitudinal homogenous wall thickening of the left common carotid artery up to 2 mm, at the junction with the axillary artery there is considerable wall thickening with sound deletion and monophasic signals in the brachial artery and the forearm, vasculitic involvement of the axillary artery is pronounced on both sides. 06/18 On the left side, continuity of the vessel of the left axillary artery can now be demonstrated and on the right side, an occlusion with strong collateralisation.	6/17 Intramural inflammatory infiltrates consistent with diagnosis of temporal arteritis plus giant cells
Patient 3, 72/♂	Worsening of the general condition, poor performance, fatigue, tiredness	08/17	n/a	n/a
Patient 4, 80/♀	Headache, polymyalgia rheumatica (myalgia of shoulder and pelvis), heavy legs, sweating, dizzy sensations when changing position	04/16	n/a	04/16 Classical for temporal arteritis
Patient 5, 71/♂	Myalgias and muscle weakness in the proximal pelvic muscles and later in the shoulders, headache in both temples, loss of appetite, weight loss, fatigue, tiredness	08/15	08/15 No ‘Halo sign’ at the temporal artery, but intermittent wall thickening	08/15 Classical for temporal arteritis
Patient 6, 83/♀	Fall and unconsciousness, headache, deterioration of general condition, weight loss, reduced performance, right auricular pain	08/10	08/10 ‘Halo sign’ right auricular	08/10 Classic signs of temporal arteritis in the right superficial temporal artery
Patient 7, 67/♀	12/16 Polymalgia Rheumatica (myalgias in shoulders and pelvis) 6/21 stabbing pain in right lower jaw and headache	06/21	06/21 ‘Halo sign’ at the temporal artery 03/23 No ‘Halo sign’, no other signs of vasculitis	07/21 Giant cells and lymphohistiocytic inflammation and incipient fibrinoid vessel wall necrosis

Patient, age (years), sex (♀, ♂)	PET	MR-angiography
Patient 1, 82/♀	n/a	11/12 High grade suspicion of dissection of the left vertebral artery with only slight flow, fresh punctate infarction in the dorsal pons at the level of the upper paramedian cerebellar peduncle on the right. 4/13 Thoracic and abdominal aorta evidence of aortitis, findings appear to continue into the superior mesenteric artery 07/13 Normal caliber aorta, no suspicious enhancement
Patient 2, 81/♀	06/17 Large vessel vasculitis in the area of the aorta, as well as the supra-aortic branches 10/17: Known large vessel vasculitis in the entire course of the aorta as well as the supra-aortic branches and the popliteal artery on both sides	n/a In the thoracic aorta, contrast medium-receiving vessel wall of the descending aorta and the subclavian artery in the proximal part on both sides as well as the left common carotid artery 11/17 Contrast-enhancement of vessel wall of the descending aorta and the proximal part of the subclavian artery on both sides as well as the left common carotid artery in known giant cell arthritis
Patient 3, 72/♂	08/17 Markedly thickened wall of the thoracic aorta and proximal abdominal aorta, supra-aortic branches, especially brachiocephalic trunk 12/17 Regredient aortitis in the supra-aortic branches, but still detectable residual inflammatory activity	08/17 Signs of severe hilar chronic aortitis of the aortic arch to the proximal abdominal aorta 12/17 Constant pronounced chronic impinging aortitis of the aortic arch to the proximal aorta abdominalis, emphasis at the aortic arch and proximal aorta abdominalis 07/18 Persistent signs of florid aortitis of the thoracic aorta 07/19 No significant wall thickening of the abdominal aorta
Patient 4, 80/♀	07/16 Inflammatory activity of the femoral and popliteal arteries, suspected minor peripheral vasculitis in femoral and popliteal pathways and a typical pattern of findings of polymyalgia rheumatica	07/16 No evidence of florid inflammation in thoracic and abdominal aorta
Patient 5, 71/♂	08/15 Increased vascular metabolic activity, consistent with large-vessel vasculitis (aorta, supra-aortic branches including subclavian arteries as well as arterial vessels of the leg) 02/16 Significant decrease in metabolic activity with evidence of continued moderate activity in the supra-aortic vessels on both sides and the aorta up to kidney level 07/16 Further decrease in inflammatory activity	12/16 Constant thickening of the vessel wall with a slight decrease in contrast medium uptake of the aortic wall, possibly to be interpreted as a response to therapy 04/17 Contrast affinity slightly decreasing 11/17 Continued regredient contrast affinity of the aortic wall
Patient 6, 83/♀	n/a	08/11 Pronounced signs of cerebral microangiopathy 05/16 No indication of active vasculitis 09/22 No indication of active vasculitis
Patient 7, 67/♀	07/21 No evidence of large vasculitis, no evidence of giant cell arteritis	n/a

Patient, age (years), sex (♀, ♂)	CRP in mg/L			Relapse	Course of therapy	Remission
	Therapy start with MMF	3 months after therapy start with MMF	6 months after therapy start with MMF			
Patient 1, 82/♀	10.8	1.2	1.3	02/16 under MTX	12/12 Prednisolone 04/13–08/13 Cyclophosphamide 8/13–01/16 MTX 1/16–03/16 Azathioprine 5/16–09/22 MMF	Since 09/22
Patient 2, 81/♀	3.9	2.7	2.2	no	06/17–02/20 Prednisolone 07/17–10/17 Cyclophosphamide 11/17–02/22 MMF	Since 02/22
Patient 3, 72/♂	28.1	0.5	0.4	no	Since 08/17 Prednisolone 08/17–09/18 MMF	Since 09/18
Patient 4, 80/♀	14.2	7	3.7	08/16 under MTX	12/14 Prednisolone 7/16–08/16 MTX 08/16–08/19 MMF	Since 08/19
Patient 5, 71/♂	36.7	4.7	0.8	no	08/15–2016 Prednisolone 10/15–03/16 MTX 03/16–07/21 MMF	Since 07/21
Patient 6, 83/♀	7.5	0.7	0.5	04/18 under MTX	Since 08/10 Prednisolone 08/10–11/10 Cyclophosphamide 12/10–05/18 MTX 05/18–07/22 MMF/ Mycophenolic acid (Myfortic)	Since 07/22
Patient 7, 67/♀	14.8	7.8	9.3	no	01/17 Prednisolone Since 07/21 Prednisolone 07/21–08/22 MTX Since 08/22 MMF/ Mycophenolic acid (Myfortic)	Since 11/22

n/a, not available; MTX, Methotrexate; MMF, Mycophenolate mofetil.

### Definition of disease activity and remission

According to the GiACTA study (9) active disease was defined as the presence of clinical signs and symptoms attributable to GCA and increased levels of inflammatory markers (CRP ≥1 mg/dL).

Remission was defined as the absence of signs and symptoms of GCA and normalization of CRP (<1 mg/dL) for more than 6 months. We extended the definition and included imaging findings, so patients were not allowed to show any activity in an imaging diagnostic.

### Data extraction

Age, sex, clinical presentation, time of diagnosis, course of therapy and time since remission were extracted from the patient record. In addition, inflammatory parameters in the blood at 3 time points (therapy start with MMF, 3 months after therapy start with MMF, 6 months after therapy start with MMF) were collected from the laboratory data. Furthermore, various radiological findings (duplex sonography, ^18^F-FDG PET, MRI) and histopathological findings (biopsies of the temporal artery) were extracted from the electronic patient record.

## Results

Seven adult patients (five women and two men) aged between 67 and 83 years (mean age 77 years) with GCA presented with differing clinical symptoms. The most common symptoms were general fatigue, general feeling of sickness and headache (*n* = 5). Two patients described visual disturbances and four patients myalgias. All patients in this case series showed inflammatory activity of the arterial vessels when diagnosed with GCA. Duplex sonography was performed at diagnosis in most of the patients. Three patients showed a classic ‘halo sign’ in one or both temporal arteries. One patient showed arterial occlusion of the femoral artery on the right and on the left side.

**Figure 1:**
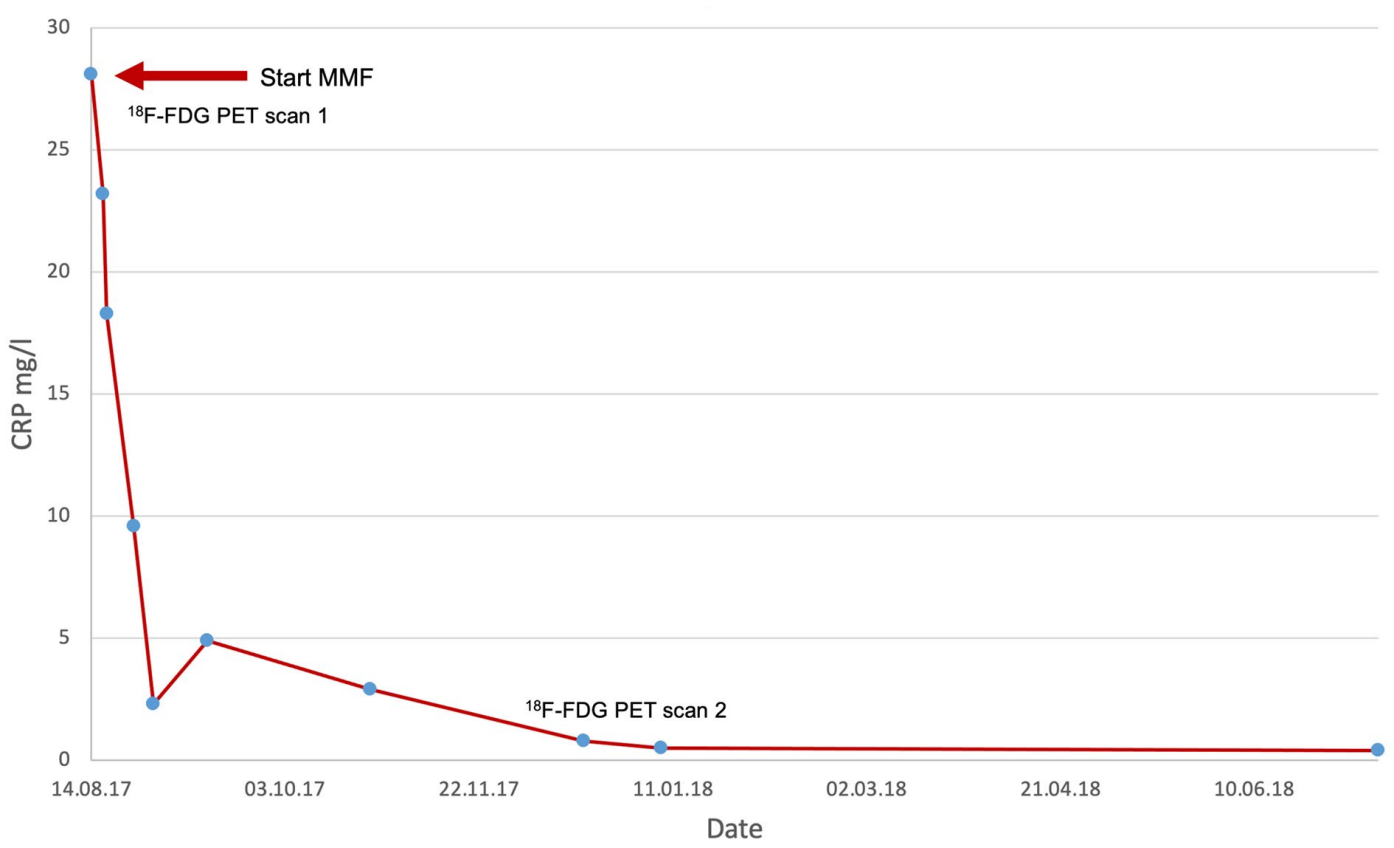
CRP values at the beginning of therapy with MMF and in the course of therapy.

Temporal artery biopsy was taken in five patients to confirm the diagnosis. A classic finding of temporal arteritis was found in all patients. Six patients were examined by MR angiography as part of the diagnostic process. Aortitis was seen in four patients. One patient showed pronounced signs of cerebral microangiopathy while no inflammation was seen in one patient on MR angiography. ^18^F-FDG PET scans were performed in five patients. Here, large vessel vasculitis was seen in the area of the aorta (*n* = 3), and lower extremity arteries (*n* = 1). In one patient, no activity was seen in the ^18^F-FDG PET scan. Two patients underwent a baseline ^18^F-FDG PET scan at time of start of therapy with MMF and a follow-up ^18^F-FDG PET scan during the course of therapy. Here, a marked reduction in inflammatory activity could be seen, consistent with response to therapy (see also Figure 1) (Detailed description is given in Table 1). The most frequent therapy before starting therapy with MMF apart from glucocorticoids was MTX (*n* = 5). During therapy with MTX 3 patients relapsed.

High dose glucocorticoid therapy (40–60 mg/day prednisone-equivalent) was initiated immediately for induction of remission in active GCA. Once disease was controlled, the GC dose was tapered to a target dose of 15–20 mg/day within 2–3 months and after 1 year to ≤5 mg/day. In case of relapse under methotrexate, the glucocorticoid dose was increased to a dose at which CRP had normalized. We recommended adjunctive therapy for all patients, since there was an increased risk of glucocorticoid-related adverse events or complications.

Mycophenolate mofetil was generally administered with a starting dose of 2 × 500 mg a day. After patients had no side effects, the dose was increased up to 3 × 500 mg a day. Two to three years after starting treatment, MMF was tapered to 2 × 500 mg, after 6 months to 2 × 250 mg, and MMF was discontinued individually. Patient 7 was started on MMF about 18 months ago and is still on therapy. In two of the patients (patient 6 and patient 7) MMF had to be switched to Mycophenolic acid (Myfortic) early in the course of therapy due to gastrointestinal symptoms such as diarrhea.

CRP levels (mg/l) of all patients decreased at the measurement time points 3–6 months (median 1.9, min 0.5, max 7), and 6–9 months (median 1.0, min 0.4, max 3.7) after initiation of therapy with MMF compared with CRP levels before MMF therapy (median 12.5, min 3.9, max 36.7). All patients with GCA tolerated MMF without major side effects. All the patients in this case series achieved disease remission. Among them, 6 patients were able to stop the therapy and are still in remission. On average, patients have been in remission for 25 months (min. 8 months, max. 58 month). One patient is still on MMF therapy today.

## Cases

### Patient 1

A 82-year-old woman first presented in 2012 with shortened walking distance, exertion-dependent pain in calves and swelling of right thigh since 2010. Furthermore, she described a general state worsening and double vision when looking to the left. Duplex ultrasonography in November 2012 revealed a femoral-type arterial occlusion with moderate-grade stenosis of the distal superficial femoral artery on the right and higher-grade stenosis in the same place on the left. MRI of the head and neck revealed a high-grade suspicion of left vertebral artery dissection with only minimal flow, as well as fresh punctate infarction in the dorsal pons at the level of the superior cerebellar peduncle paramedian on the right. The patient was treated with prednisolone 60 mg and then cyclophosphamide. She subsequently received methotrexate as baseline medication. The patient then relapsed in November with mildly elevated CRP (10 mg/L). After that she received MMF in February 2016 until September 2019. This resulted in a decrease in CRP levels (see Table 1, summerizes all patients, please refer to the table after the last patient). MMF could be stopped in 2019. The patient has been in remission since then.

### Patient 2

A 81-year-old woman presented in June 2017 with unilateral left-sided headache, intermittent visual disturbances, reduced general condition and weight loss. She also had claudication of the upper extremities. Attempts to measure blood pressure in the upper extremities failed. A duplex ultrasonography of the cerebral arteries showed a ‘halo sign’ at the exit side of the left temporal artery, preauricular, and in the more posterior region of the branch of the temporal artery and occlusions of the axillar arteries. Two ^18^F-FDG PET scans in 2017 revealed large-vessel vasculitis throughout the aorta as well as the supra-aortic branches and popliteal arteries. MRI showed arterial wall contrast enhancement of the descending aorta, the subclavian artery in the proximal part on both sides, and the left common carotid artery. A biopsy of the temporal artery in 2017 showed intramural inflammatory infiltrates plus giant cells consistent with a diagnosis of temporal arteritis. The patient was treated with one cycle of cyclophosphamide followed by MMF from 2017 to 2022. The patient has been in remission for 16 months.

### Patient 3

**Figure 2:**
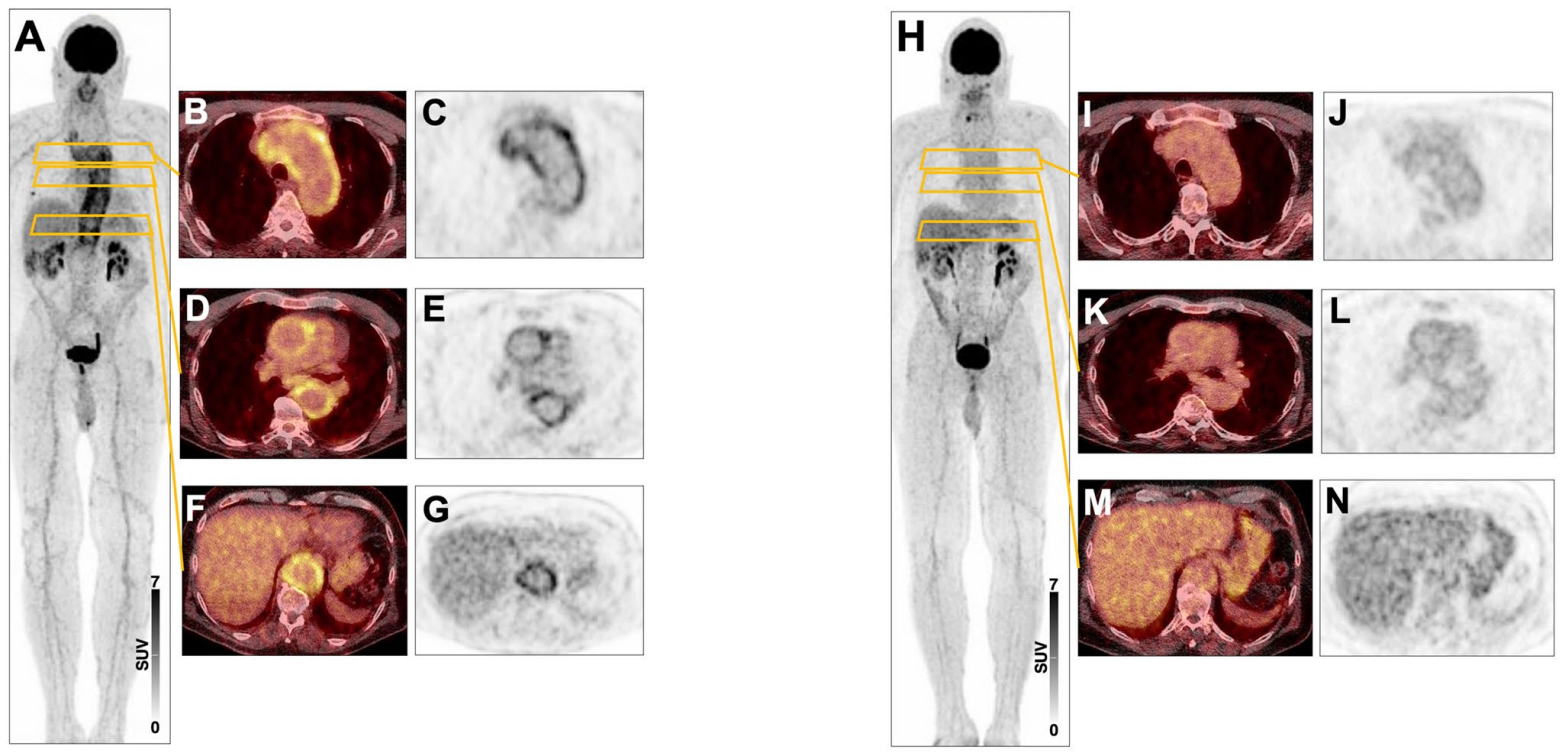
**(A)** 08/17 Maximum-intensity projection (MIP) ^18^F-FDG PET image showing active large vessel vasculitis in a 3-year-old male patient. **(B–G)** Transversal fused PET/CT and PET images showing high inflammatory arterial wall signal in the aortic arch **(B,C)** and the thoracic **(D,E)** and abdominal aorta **(F,G)**. **(H–N)** 12/2017 Corresponding PET images after treatment showing markedly reduced signal within arterial walls.

A 72-year-old man first presented in 2017 with worsening general condition, lack of performance, exhaustion and fatigue. ^18^F-FDG PET at the time of diagnosis showed increased metabolic activity and a markedly thickened wall of the thoracic aorta and proximal abdominal aorta, as well as in the supra-aortic branches, particularly in the brachiocephalic trunk bilaterally. At diagnosis, the patient was receiving glucocorticoids and MMF. CRP levels dropped from 28.1 mg/L at diagnosis to 0.5 mg/L after 3 months of MMF treatment (see Figure 1). ^18^F-FDG PET 1 year later, after discontinuing MMF, showed a marked signal (see Figure 2). The patient has been in remission for 3.5 years.

### Patient 4

A 80-year-old woman first presented in 2014 with headache, polymyalgia rheumatica (myalgias in the shoulders and pelvis), heavy legs, heavy sweating and vertigo when changing position. ^18^F-FDG PET in 2016 showed marked inflammatory activity in the femoral and popliteal arteries with a suspicion of minor peripheral vasculitis in femoral and popliteal stromal pathways. A biopsy showed classic finding for arteritis temporalis. At time of diagnosis the patient received 75 mg prednisolone, then as a baseline medication of 15 mg methotrexate (MTX). Relapse occurred while trying to reduce prednisolone. Therefore, MTX was stopped and therapy with MMF was started. This resulted in a remission, so that MMF could be discontinued in 2019. Clinical remission has been achieved for 2.5 years.

### Patient 5

A 71-year-old man first presented in 2015 with myalgias and muscle weakness in the proximal pelvic muscles and later in the shoulder girdle, headache in both temples, loss of appetite (weight loss of 7 kg within 1 year) fatigue and tiredness. Ultrasound showed no ‘halo sign’ at the temporal arteries, but intermittent wall thickening. ^18^F-FDG PET in 2015 showed increased vascular metabolic activity, consistent with large-vessel vasculitis. Vasculitis signs were accentuated in the aorta, the arteria subclavian in both sides and the supra-aortic branches as well as in the in the lower extremities. A biopsy in 2015 showed classic finding for arteritis temporalis.

After diagnosis, the patient received MTX and Prednisolone until 2016 when he showed elevated transaminases. He then was switched to MMF, the therapy could be discontinued in 2021. The patient developed steroid-induced diabetes mellitus type 2 during the course of the disease.

Two follow-up^18^F-FDG PET scans after treatment start with MMF in 2016 showed significant decrease in metabolic activity of large vessel vasculitis with evidence of continued moderate activity in supra-aortic vessels bilaterally and aorta.

Clinical remission and normalization of the laboratory parameters have been achieved under MMF for 2 years.

### Patient 6

A 83-year-old woman first presented in 2010 with a fall with unconsciousness, headache, worsening of general condition, weight loss, reduced performance and right auricular pain. A duplex ultrasonography of the cerebral arteries showed a ‘halo sign’ right auricular. A biopsy of the right superficial temporal artery showed classic signs of temporal arteritis. In 2011 an MRI was conducted and showed pronounced signs of cerebral microangiopathy. The patient was given MTX in 2010–2018. In 2018, the patient had a relapse with retro auricular pain on the left and mildly elevated CRP levels. The therapy was thus changed, therapy with MMF was initiated. Clinical remission has been achieved for 1 year.

### Patient 7

A 67-year-old woman was diagnosed with polymyalgia rheumatica (myalgias in shoulder and pelvis) in 2016 and treated with MTX and Prednisolone. In 2021 the patient had a glucocorticoid-induced complicated urinary tract infection. In 2022, the patient developed achillodynia under MTX. In 2021 she presented with stabbing pain in the right lower jaw and headache. A ‘halo sign’ was detected at the right temporal artery in an ultra sound. ^18^F-FDG PET showed no evidence of large vasculitis and no evidence of giant cell arteritis. However, a biopsy revealed giant cells and lymphohistiocytic inflammation as well as incipient fibrinoid vessel wall necrosis. The patient was changed to MMF, which she is still given. CRP has decreased, but is still slightly elevated. This is due to active furunculosis, the patient suffers from as a comorbidity. As far as her vasculitis is concerned, she is still in remission. An overview of demographic data, symptoms, diagnostics and therapy for all patients is provided in Table 1.

## Discussion

We observed 7 patients with giant cell arteritis who were treated with MMF, mostly after relapse of the disease under medication with MTX (*n* = 3). Most of the patients tolerated MMF well without any adverse drug reactions, matching safety signals from other studies (7, 8). Two patients developed diarrhea under MMF, so they were switched to Mycophenolic acid (Myfortic). During the treatment with MMF no severe adverse events were observed. Our study presents a very heterogeneous group of patients regarding the constellation of symptoms in giant cell arteritis involving the extracranial vessels with typical arteritis temporalis, but also involving the thoracic and abdominal aorta and the arteries of the upper and lower extremities, which illustrates the difficulty of finding a diagnosis and the existence of subgroups of different phenotype (10).

Untreated large-vessel vasculitis can lead to serious complications such as blindness or stroke (11). Temporal artery biopsy has been the preferred diagnostic method for GCA, but its sensitivity is limited by sample quality and investigator skills (12, 13). Diagnostic imaging, such as ultrasound and MRI, is gaining importance and is part of the EULAR recommendations, with ultrasound being the favored method for cranial GCA, and MRI being equivalent but less accessible. MRI, CT, and ^18^F-FDG PET can be used for diagnosing extracranial GCA, whereas ^18^F-FDG PET is particularly useful in differentiating vasculitis from other conditions (14, 15). In our case series, imaging techniques were used in all patients to establish the diagnosis and in most cases a biopsy of the temporal artery was performed. To confirm the diagnosis and to exclude differential diagnoses in case of nonspecific clinical symptoms such as B-symptoms, we predominantly used ^18^F-FDG PET. Imaging techniques were used to determine the extent of vascular involvement and to objectify the response to therapy with MMF. Furthermore, dangerous events could be averted, such as dissections, through early detection.

In addition to imaging methods, we used laboratory parameters to assess disease activity. Although not a specific marker for the diagnosis of giant cell arteritis, serum CRP is commonly used for disease monitoring during treatment (16). In 2017, the multicenter phase III GiACTA trial showed a benefit of tocilizumab in sustained remission versus placebo with comparatively significant steroid sparing and defined remission by normalization of CRP levels in the absence of clinical symptoms for disease activity. Supportive imaging techniques were not used (17). All patients of our case series had elevated CRP levels at the time of diagnosis and CRP levels decreased after starting medication with MMF until normalization. Only in one patient serum CRP did not normalize due to an active furunculosis, which confirms that this biomarker can still be utilized for disease monitoring and detecting severe infections. We also considered normalization of CRP as remission and in most patients the dynamics of the CRP serum level could be correlated with a reduction of disease activity in the imaging used during the course (*n* = 6). This is also how remission was confirmed in the patient with furunculosis.

Steroids are the basic therapy for giant cell arteritis and are used over a long time. Recent studies on ANCA vasculitis visualized the undesirable side effects of steroid toxicity by using the Glucocorticoid Toxicity Index and to show positive effects by steroid sparing (18). Currently, tocilizumab is the only approved and effective therapeutic option for GCA making steroid sparing possible. Tocilizumab acts via interleukin-6 blockade resulting in a reduction of acute phase proteins such as CRP in serum. During therapy with Tocilizumab, CRP can no longer be used reliably as an indication of disease activity or relapse of giant cell arteritis (19), which emphasizes the importance of the additional use of imaging techniques. This also indicates that CRP cannot be utilized as a reliable marker of severe bacterial infection in these patients (20), and in contrast to the GiACTA trial, a multicentre study in patients with GCA given tocilizumab reported far more serious infections (11.9%) (21).

The withdrawal of CRP as a monitoring parameter and the fact that relapses were observed in up to 30% of patients treated with tocilizumab, indicate the need for additional treatment options (9).

Considering the pathogenesis of giant cell arteritis and the already existing experience with MMF in remission induction in ANCA vasculitis (5), the use of MMF in giant cell arteritis seems obvious. To our knowledge, there are two other case series besides ours that investigated MMF in GCA in the past. Sciascia et al. (7) showed in 3 patients that MMF may be considered a steroid-sparing agent in elderly patients with GCA. The second case-series included 37 patients retrospectively suffering from GCA with large vessel involvement who were treated with MMF. After 2 years, most of the patients (*n* = 31) remained on MMF, whereas 6 had switched to MTX or tocilizumab due to relapse (8). In line with the results of these case series, that showed that MMF is effective in controlling disease activity and reduces corticosteroid dosage, all patients in this case series achieved remission on medication with MMF; some patients showed sustained remission even years after end of treatment. Since we did not assess the cumulative steroid dosages, we cannot make any further statement on steroid sparing, even if steroids could be reduced in all patients.

Especially in patients with GCA refractory to treatment with disease modifying antirheumatic drugs (DMARDs), MMF could be an important alternative. Economically, MMF also represents a more cost-effective alternative to tocilizumab. Since MMF potentially exhibits less severe toxic effects than MTX, the effectiveness of MMF in GCA should be investigated in future randomized studies. These results should be considered in future treatment guidelines.

## Conclusion

The results of our case series suggest that MMF could be an effective additional therapeutic option for the treatment of GCA considering remission induction and sustained remission. Our cohort presented a very heterogeneous group of patients including the classic pattern of cranial GCA and large-vessel GCA (10). Remission was induced independent of the phenotype of GCA. In addition to serum CRP levels, diagnosis and monitoring of disease activity was accompanied by extensive imaging techniques, leading to more safety in the assessment of disease activity and remission.

## Data availability statement

The raw data supporting the conclusions of this article will be made available by the authors, without undue reservation.

## Ethics statement

Ethical review and approval was not required for the study on human participants in accordance with the local legislation and institutional requirements. Written informed consent from the [patients/participants OR patients/participants legal guardian/next of kin] was not required to participate in this study in accordance with the national legislation and the institutional requirements.

## Author contributions

AP: Formal analysis, Visualization, Writing – original draft. SS: Writing – original draft. TD: Methodology, Visualization, Writing – review & editing. MH: Writing – review & editing. AW: Conceptualization, Methodology, Writing – review & editing.
